# SiNWs Biophysically Regulate the Fates of Human Mesenchymal Stem Cells

**DOI:** 10.1038/s41598-018-30854-3

**Published:** 2018-08-27

**Authors:** Hsin-I Lin, Shu-Wen Kuo, Ta-Jen Yen, Oscar K. Lee

**Affiliations:** 1 Department of Materials Science and Engineering, National Tsing Hua University, Hsinchu, Taiwan; 2 Department of Medical Research, Taipei Veterans General Hospital, Taipei, Taiwan; 3 Stem Cell Research Center, National Yang Ming University, Taipei, Taiwan; 4 Frontier Research Center on Fundamental and Applied Sciences of Matters, National Tsing Hua University, Hsinchu, Taiwan; 5 High Entropy Materials Center, National Tsing Hua University, Hsinchu, Taiwan; 6 Department of Orthopaedics and Traumatology, Taipei Veterans General Hospital, Taipei, Taiwan; 7 Taipei City Hospital, Taipei, Taiwan

## Abstract

While biophysical stimuli from polymeric matrices are known to significantly affect the fates of human mesenchymal stem cells (hMSCs), the stimulatory effects of nano-sized silicon-based matrices on hMSCs have not been thoroughly investigated. We previously demonstrated that vertically aligned, single-crystalline silicon nanowires (SiNWs) can control the osteogenicity of hMSCs via controllable spring constants from SiNWs matrix. However, other possible differentiation fates of hMSCs on SiNWs have not been explored. We hypothesize that tunable spring constant from artificial SiNWs matrices can direct different types of hMSC differentiations. The spring constants of tunable SiNW matrices can be consistently controlled by tuning the SiNW length. The results of gene expression and cell stiffness suggest that hMSCs differentiations are sensitive to our distinguishable spring constants from the SiNWs groups, and simultaneously conduct osteogenicity and adipogenicity. These findings suggest that SiNW matrices can regulate the fates of hMSCs when the SiNW characteristics are carefully tuned.

## Introduction

Regenerative medicine is a multidisciplinary field that combines biology, materials science and engineering, and mechanical design to ameliorate complex diseases, physical imperfections, and disorders in humans. Self-renewing and multipotent stem cells are ideal for treating such complicated conditions. Multilineage stem cells that are typically collected from bone marrow, umbilical cord tissue, and placenta, are indispensable to artificial tissue engineering^1–4^ and neuroregeneration^5–7^. Before the full potential of stem cell therapy in artificial tissue engineering can be attained, it is necessary to develop precise approaches to manipulate stem cell fates^8^.

To cure physiological problems such as organ failure^8,9^ and type I diabetes^10^ using hematopoietic stem cells^8^, stem cell fates must be precisely controlled. However, the desired therapeutic effects can be achieved only by using stem cells that undergo specific transitions resulting from complex induction factors and stimuli from microenvironments. Biophysical and biochemical stimuli are two common means to direct the stem cell fate transitions. Biophysical stimulation involves elasticity of polymeric substrates^11–13^, electric-field induction^14^, and photostimulation^15^, whereas biochemical stimulation is primarily achieved via growth factors^16,17^, protein mediation^18^, and drug carriers^19^.

Regulation pathways and types of stimuli strongly affect stem cell fates. Elasticity of a flat polymeric matrix^11–13^ is one of the most straightforward methods of biophysical stimulation for manipulating stem-cell fate. Several studies have demonstrated that mesenchymal stem cell (MSC) fates are affected by the elasticity^13,20^ and topography of the extracellular matrix^21^. Moreover, osteogenesis and adipogenesis are favored by stiff and flexible matrices, respectively^12,22^. While the relationship between stem cell fate transition and the elasticity of flat culture plates has been evaluated, little is known about the effects of silicon nanowires (SiNWs) on stem-cell differentiation and variations in cell stiffness. We evaluated the effects of SiNW stiffness (spring constant, *K*, determined theoretically and by *in situ* measurements)^23^ on the differentiation of human MSCs (hMSCs) stimulated by SiNW matrices and the distributions of hMSC stiffness after differentiation. The SiNW matrix is an excellent platform for evaluating how extracellular stimulation from matrices of various stiffnesses, mechanotransduction, and microenvironment affect stem-cell fate. The ultimate goal is to profile a map of hMSC differentiation with regard to SiNWs stimulations for use in clinical applications.

First, based on theoretical calculations of *K* using beam theory^24^ and *in situ* nano-indentation measurements^25,26^, we evaluated the consistency between the theoretical and experimental values of *K* and investigated the effects of SiNW dimensions on the mechanical properties of SiNWs groups. Subsequently, hMSCs were cultured on the SiNWs groups to evaluate cell fate after differentiation. Finally, we mapped elasticity distributions of the fixed and living hMSCs that adhered to the SiNWs. Based on the above evaluations, we analyzed the correlations among SiNW dimensions, hMSC fate regulation, and mechanical properties.

## Stiffness of SiNWs groups

**Figure 1:**
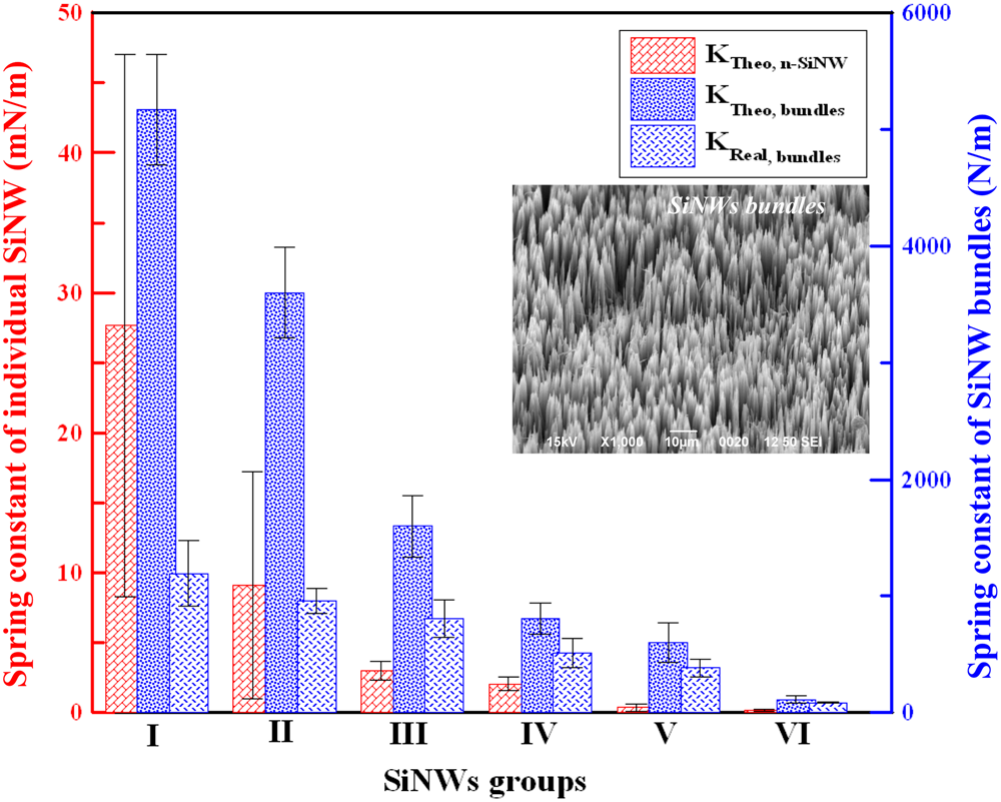
Spring constants of individual SiNWs and SiNW bundles obtained by beam theory and *in situ* TEM indentation. The theoretical spring constants of individual SiNWs and SiNW bundles in the six SiNWs groups were calculated using beam theory [Equation (1)] from the SiNW dimensions (*L* and *D*). At least 300 individual SiNWs were analyzed for each group. The spring constants of SiNW bundles were also determined on the basis of force–displacement curves measured by *in situ* TEM picoindentation (PI 95 TEM Picoindenter, Hysitron, USA). The TEM image in the inset (JEM 2010, Jeol, Japan) shows that the SiNWs easily formed bundles because of capillarity (stiction).

In our previous study, we designed six SiNWs groups, according to SiNWs preparation time, to generate tunable spring constants. SiNWs Group I, the shortest SiNWs group, regulated osteogenic differentiation in hMSCs^23^. An idea that can other SiNWs groups direct the fates of hMSCs appeared. Therefore, in this study, we attempted to identify stem cell fates that can be controlled using different SiNWs groups. We fabricated vertically aligned, dense, and length-controllable SiNW arrays^23,27^ as cell-culture matrices on single-crystalline Si (100) chips using electroless metal deposition (EMD). In the EMD process, silver nanoparticles (AgNPs) in an aqueous silver nitrate solution [AgNO_3_(aq)] served as the oxidizing agent to form SiO_x_ nanospots. Upon etching with fluorine ions, these SiO_x_ nanospots generated vertical pits because of the anisotropic etching behavior of orientated Si chips. EMD was performed under a constant concentration of electrolyte solution [0.03 M AgNO_3_(aq) + 4.6 M HF(aq)] and fixed temperature at 50 °C ± 1 °C, and different etching periods (5–60 min) were applied to prepare six groups of dense SiNW arrays with various dimensions (Table S1). These fabrication conditions produced a series of SiNWs with different *K*, which served as the dominant source of biophysical stimulation in this work. The etching process that was used is stable and achieves a constant etching rate of 1.06 μm/min^23,27^. For each SiNWs group, the average diameter (*D*_1_) and length (*L*_1_) were determined on the basis of 300 individual SiNWs, and the theoretical value of *K* (*K*_Theo,SiNW_) was calculated using beam theory^24^ [Equation (1); Table 1 and Fig. 1]:

1 $$K=\frac{{D}^{{\rm{4}}}E}{4{L}^{3}},$$

where *E* is the Young’s modulus of (100) Si (*E* = 170 GPa), *L* is SiNW length, and *D* is the SiNW diameter. During the EMD process^23,27^, etching period was proportional to *L*_1_ because of anisotropic etching behavior. *D*_1_ remained within a small range of 160–190 nm. (100)-orientated single-crystalline Si and anisotropic etchants, AgNPs and fluorine ions, both settled the uniform and directional etching reaction^23,27^.

**Table 1:** Spring constants of the six SiNW groups.

	*L*_1_ (μm)	*D*_1_ (nm)	*K*_Theo,SiNW_ (mN/m)	*D*_2_ (μm)	*K*_Theo,bundles_ (N/m)	*K*_Real,bundles_ (N/m)
I	8.7 ± 0.4	162.3 ± 33.1	44.0 ± 31.0	3.3 ± 0.21	5,170 ± 470	1,200 ± 280
II	13.5 ± 0.4	170.6 ± 43.5	15.0 ± 13.0	3.8 ± 0.47	3,600 ± 390	960 ± 110
III	20.2 ± 2.2	174.7 ± 23.9	4.8 ± 1.1	4.2 ± 0.36	1,600 ± 265	810 ± 160
IV	25.9 ± 4.0	191.7 ± 34.4	3.3 ± 0.8	4.7 ± 0.82	808 ± 132	520 ± 110
V	34.0 ± 0.7	154.0 ± 25.1	0.6 ± 0.4	4.9 ± 0.49	602 ± 167	380 ± 70
VI	63.5 ± 3.5	190.1 ± 25.7	0.2 ± 0.08	5.1 ± 0.74	112 ± 32	86 ± 4

*L*_1_: Average length of individual SiNWs; *D*_1_: Average diameter of individual SiNWs; *D*_*2*_: Average diameter of SiNW bundles; *K*_Theo,SiNW_: Theoretical spring constants of individual SiNWs computed by beam theory from *L*_1_ and *D*_*1*_; *K*_Theo,bundles_: Theoretical spring constants of SiNW bundles computed by beam theory from *L*_*1*_ and *D*_2_; *K*_Real,bundles_: Real spring constants of SiNW bundles measured by *in situ* TEM picoindentation.

Interestingly, the SiNWs uniformly synthesized on (100) Si chips easily formed the micro-bundles because of capillary action or stiction^28^. While stiction is a drawback of microelectromechanical systems (MEMS), the assembled SiNW micro-bundles are important for delivering mechanical stimuli to hMSCs. Each individual SiNW and SiNW bundle behaves like a mechanical spring (beam), effectively supporting hMSCs adhesion and stimulating hMSCs growth and differentiation. The inset of Fig. 1 shows a SEM image of the SiNW bundles. Notably, the longer, more flexible SiNWs aggregated with surrounding nanowires to a greater extent than did the shorter SiNWs, to form bundles with larger diameters (*D*_2_). As shown in Table 1, *K*_Theo,SiNW_ was inversely proportional to SiNW length. The same trend was observed in the spring constants of SiNW bundles (*K*_Theo,bundles_); however, the different diameter scales of the SiNWs and bundles (nm vs. μm, reflectively) affected the magnitudes of *K*_Theo,SiNW_ (mN/m) and *K*_Theo,bundles_ (N/m).

The spring constants of the SiNW bundles (*K*_Real,bundles_) were also experimentally measured using *in situ* transmission electron microscopy (TEM) picoindentation. First, a focused ion beam microscope was used to prepare SiNW samples on Cu TEM grids. In an *in situ* TEM picoindentation instrument, the SiNW bundles were indented to a depth of 400 nm using a cone-shaped flat-end tip with a 5-μm punching diameter. The force–displacement curves were recorded during indentation. Based on these force–displacement curves, the following values of *K*_Real,bundles_ were calculated for the six SiNWs groups (Table 1): 1.20 ± 0.28 (Group I), 0.96 ± 0.11 (Group II), 0.81 ± 0.16 (Group III), 0.52 ± 0.11 (Group IV), 0.38 ± 0.07 (Group V), and 0.086 ± 0.004 kN/m (Group VI). The theoretical and real spring constants of the SiNWs decreased significantly with increasing SiNW length (Fig. 1). The shortest SiNWs (Group I) were the stiffest with the highest spring constants, whereas the longest SiNWs (Group VI) were the softest with the lowest spring constants. The effect of the minor differences in diameter among the six SiNWs groups on the spring constants was weaker than that of SiNW length. However, *K* was higher for the SiNW bundles than the single SiNWs, indicating that the large diameters of the bundles (*D*_2_) enhanced the spring constants. The spring constant measurements made by nanoindentation were in agreement with those calculated by beam theory (Table 1).

### Osteogenicity and adipogenicity of hMSCs stimulated by SiNWs

We hypothesize that SiNWs matrices with different spring constants initiate a series of reactions through integrin heterodimers, focal adhesion kinase, and vinculin send stimulating messages to the nucleus that eventually regulate the fate transitions of hMSCs. Substrate stiffness has been shown to affect stem cell fates^11–13,22^. We found the stimulations from Group I SiNWs conducted to the cultivating hMSCs under maintaining medium greatly boost the osteogenicity of hMSCs^23^. In addition to osteogenicity, we investigated other fate transitions that were promoted by SiNWs groups in this study. To decode the fate regulations of SiNWs groups, we used osteogenic markers (*COL1α1* and *RUNX-2*) and adipogenic markers [*PPARγ* and fatty acid-binding protein 4 (*FABP4*)] to determine the fates of hMSCs after SiNW stimulation.

**Figure 2:**
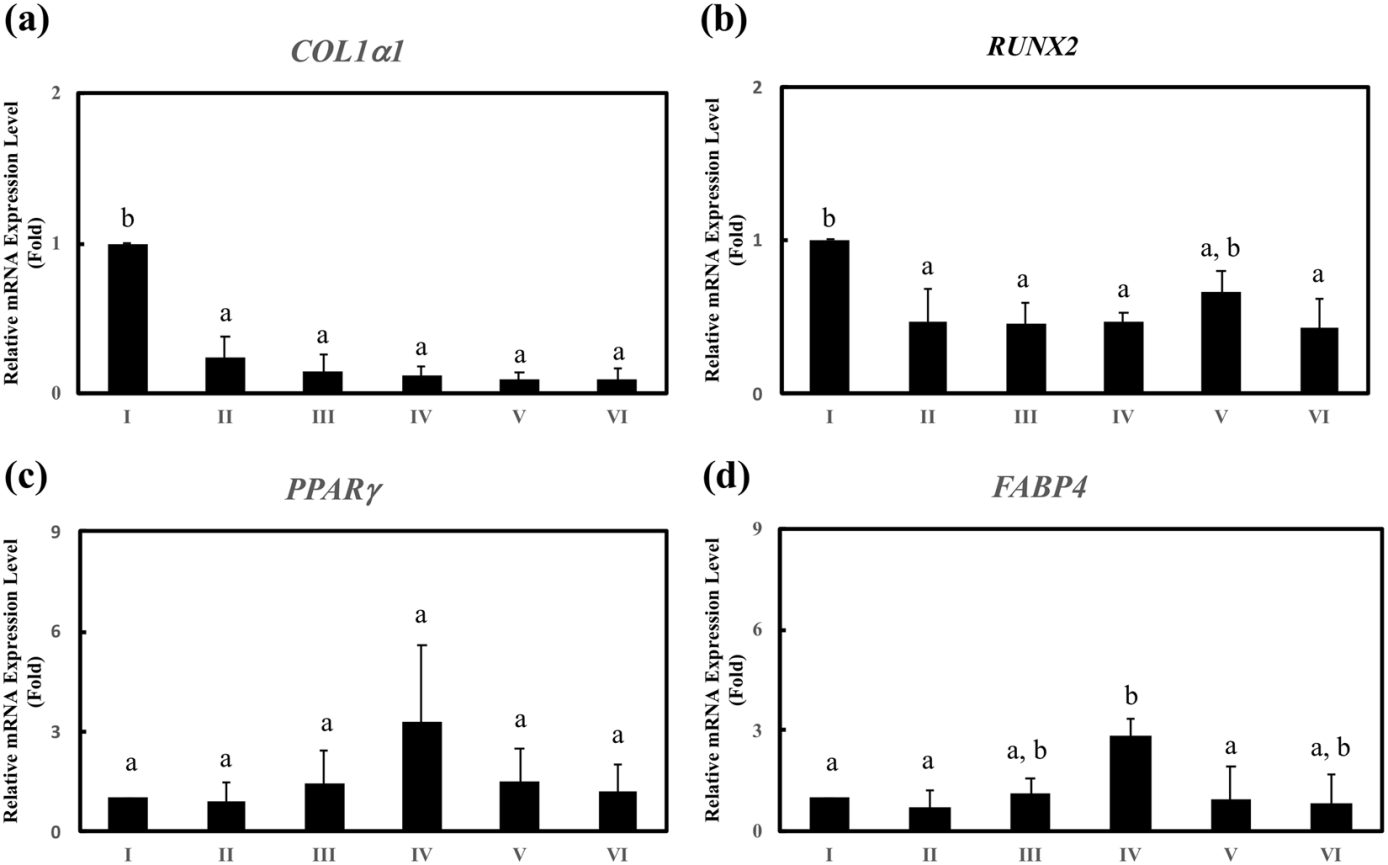
Gene expression of osteogenic markers (**a**) *COL*1*α*1 and (**b**) *RUNX-*2 and adipogenic markers (**c**) *PPARγ* and (**d**) *FABP4* after hMSCs were cultured on SiNWs for four days under adipogenic medium (**b**: *P* < 0.05). N = 3.

For osteogenicity of hMSCs, gene expressions showed the obvious trend on SiNWs groups between *COL*1*α1* and *RUNX-2* [Fig. 2(a,b)] under adipogenic medium. These gene expressions, after a series of RNA extraction and qRT-PCR amplification, quantitatively indicated that SiNWs in Group I with the highest spring constant (*K*_Real,bundles_ = 1.2 kN/m) obviously promote the *COL1α*1 and *RUNX-2* expression in adipogenic medium. *COL*1*α*1 and *RUNX-2* expressions were enhanced 10.1- and 2.4-fold, respectively, in Group I than in Group VI. Intriguingly, Group I SiNWs, under maintain medium exhibited osteogenicity that was similar to that which was observed in our previous study^5^. The statistical significance of osteogenic gene markers indicate that Group I SiNWs were strongly favorable to hMSC osteogenicity under both the maintain and adipogenic media, compared to the other SiNWs groups^5^.

The adipogenicity of the hMSCs [Fig. 2(c,d)] exhibited completely different trend on gene expression, compared to osteogenicity. Adipogenic gene expression reached a plateau on Group IV SiNWs on *PPARγ* and *FABP4*. Expression of *PPARγ* and *FABP4* was enhanced by 3.3- and 2.9-fold, respectively, in Group IV compared to those of Group I SiNWs. However, there is no statistical significance of *PPARγ* expression among the six SiNWs groups. In contrast, Group IV SiNWs obtained the statistical significance on *FABP4*. The Group IV SiNWs with moderate spring constant (*K*_Real,bundles_ = 0.5 kN/m) is favorable to adipogenicity of hMSCs. The results indicate that the SiNWs with different spring constants selectively triggered osteogenicity and adipogenicity in hMSCs. We then applied Group I, IV, and VI SiNWs to investigate the effects of spring constant on cell morphology, cell stiffness, and fate regulation.

### Cell morphology and stiffness of fixed and living hMSCs on SiNWs

**Figure 3:**
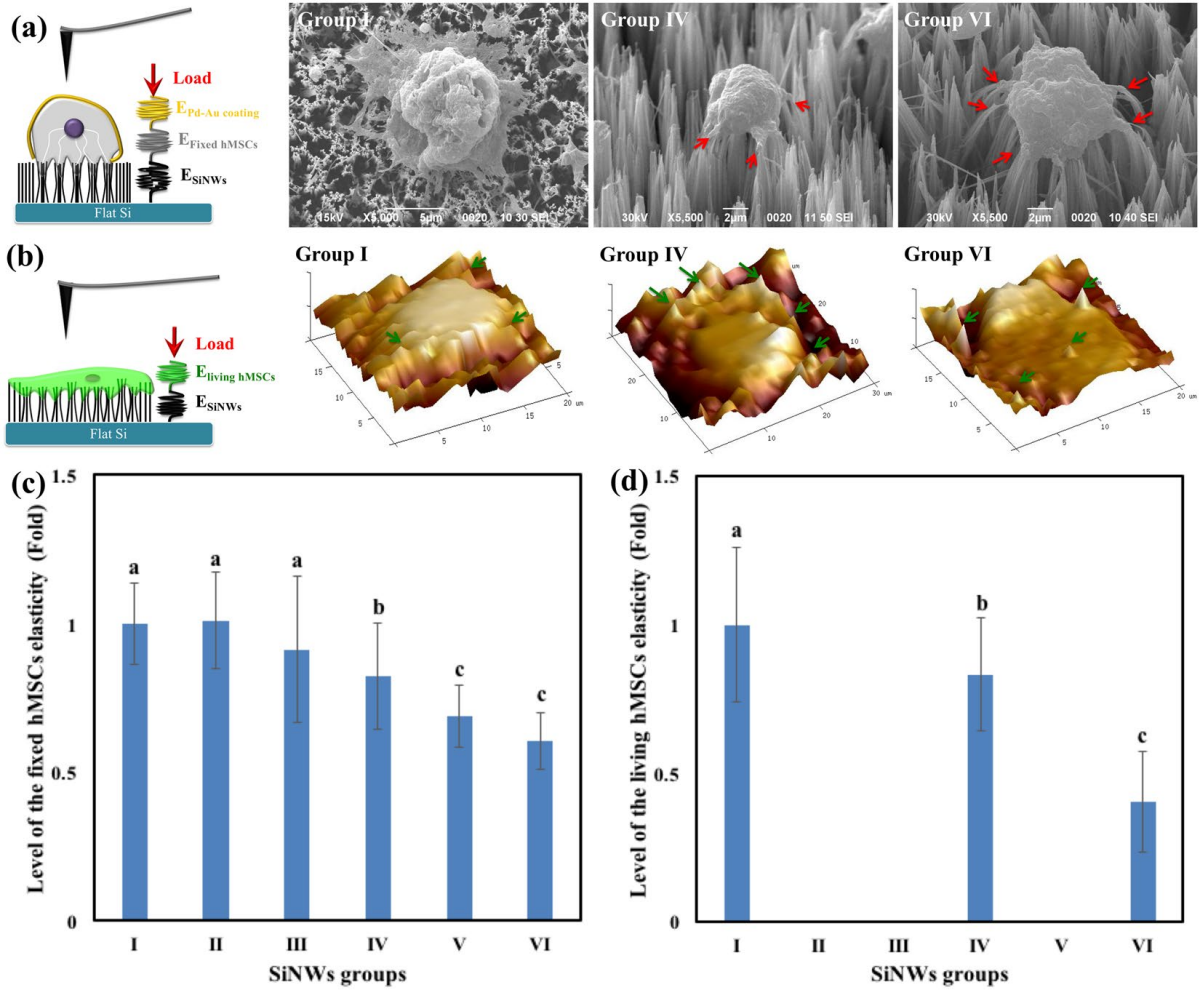
Schematic, SEM images, and cell stiffness of fixed and living hMSCs on SiNWs. Schematic showing (**a**) fixed and (**b**) living hMSCs differentiated on SiNWs. Cell stiffness of (**c**) fixed and (**d**) living hMSCs differentiated on SiNWs in PBS-containing petri dishes; the force–displacement curves were evaluated using Dimension Icon (Bruker, USA) and Bioscope Catalyst (Bruker, USA) instruments, respectively. (**b**: *P* < 0.05; c: *P* < 0.01).

The above results confirm that the SiNWs with different spring constants affected the fates of hMSCs in this study. Next, we evaluated the cell morphology and stiffness for fixed and living cells on SiNWs after four days of induced differentiation. The fixed hMSCs grown on SiNWs were dehydrated, stained, and sputtered with Pd–Au thin films prior to morphological assessment using scanning electron microscopy (SEM). The SiNWs arrays formed bundles as a result of capillarity [Fig. 3(a)], and the physical properties of the bundles determined the cell adhesion/growth behaviors^23^ [Fig. 3(a,b)]. The fixed hMSCs were observed on the tips of the SiNW micro-bundles, and sensed that SiNWs are non-cytotoxic^23,29^. The produced protrusion [red arrow in Fig. 3(a)] from hMSCs covered these surrounding SiNWs micro-bundles and received the simulations from their stiffness. Group I SiNWs, which were the shortest and produced the stiffest bundles, supported cell adhesion similar to that of flat substrate that cells grown along *X*–*Y* plane more than along *Z* direction and cell wrapped the tips of these nanowire bundles^30^. In contrast, the SiNWs in Groups IV, V, and VI formed more flexible and distinct bundles because of their greater lengths. This forced the cells to grow primarily along the *Z* direction by expanding protrusions to congregate the surrounding bundles toward the cell body^23^. The red arrows in Fig. 3(a) highlight the bent tips of SiNW bundles resulting from the contraction force generated by the hMSCs. The side-view SEM images indicate that the Group IV and VI SiNW bundles clearly penetrated the cell membrane^23^.

Second, living hMSCs grown on SiNWs in adipogenic medium after four days of cultivation were mapped by *in situ* atomic force microscope (AFM) indentation in petri dishes containing PBS [Fig. 3(b)]. In contrast to the fixed hMSCs on SiNWs, the living cells were embedded and flattened on the SiNW matrix, and SiNW bundles in Groups I, IV, and VI penetrated the cells [green arrows in Fig. 3(b)]^30^. Less cell penetration was observed for Group I SiNWs because the surfaces of these SiNWs were relatively flat, and most of the penetrating peaks occurred at the outer region of cell body. Group IV and VI SiNWs exhibited a greater degree of cell penetration and contributed around the cell body due to the distinct SiNW bundles.

Finally, we measured the stiffnesses of fixed [Fig. 3(c)] and living [Fig. 3(d)] hMSCs on SiNWs. These differentiated hMSCs on SiNWs groups should present their distinguishable mechanical properties between osteogenesis and adipogenesis^31^. The highest cell stiffness was observed for cells on Group I SiNWs, which stimulated osteogenicity in the hMSCs. In contrast, the Group IV SiNWs, which stimulated adipogenicity, resulted in the lowest hMSC stiffness. As indicated by the adipogenic gene markers in Fig. 2(c), no clear adipogenicity was observed in the hMSCs on Group V–VI SiNWs. Figure 3(c,d) indicate that cells on Group VI SiNWs had the lowest cell stiffness but did not regulate hMSCs into adipogenicity. This implies that mechanical simulation by Group VI SiNWs may have resulted in other cell fates of hMSCs. Collectively, the different SiNWs fabricated in the present study were able to direct hMSCs into osteogenicity or adipogenicity.

## Discussion

SiNWs have been shown to initiate unique mechanical stimulations and perform signal transductions through integrin heterodimers, pFAK, and vinculin to the, resulting in the successful induction of osteogenicity in hMSCs^23^. In this study, we sought other possible fates of hMSCs regulated by chemically equivalent SiNWs with different physical properties (dimensions, spring constants, and stiffness). We adopted beam theory^24^ to compute the theoretical spring constants of individual SiNWs and SiNW bundles (Table 1 and Fig. 1). When cells were cultured on SiNWs, they produced protrusions that grabbed (bent) the surrounding SiNW bundles, pulling them toward the cell body [Fig. 3(a)]. These bending SiNW bundles strongly supported cell adherence, growth, and stimulation, in accordance with beam theory.

In beam theory, each SiNW or SiNW bundles are considered to be perfect cylindrical objects without the imperfections bent with the lateral loading; however, in reality, the SiNW bundles were formed from tens of nanowires with weak capillarity (stiction force) and imperfections among the SiNWs. These geometric differences explain the observed variations between *K*_Theo,bundles_ and *K*_Real,bundles_. However, for all the samples, SiNW length was inversely related to spring constant; thus, the shortest SiNWs (Group I) exhibited the stiffest (largest) spring constant, whereas the longest (Group VI) had the softest (lowest) spring constant.

The SiNWs were employed as sources of biomechanical stimulation to induce hMSC growth and differentiation. González-Cruz *et al*. demonstrated that adipogenesis was positively correlated with cell height and negatively correlated with cell stiffness, whereas osteogenesis was positively correlated with cell stiffness^31^. A quintessential transition of osteogenicity and adipogenicity was observed in Fig. 2; the optimal levels of hMSC osteogenicity and adipogenicity were observed for the Group I and Group IV SiNWs, respectively. Thus, based on gene expression, the SiNWs were able to regulate the hMSCs into specific fates under adipogenic induction medium. Interestingly, Group I SiNWs strongly regulated the osteogenic differentiation of hMSCs under either maintain medium or adipogenic induction medium. Moreover, the Group I SiNWs resulted in the greatest cell stiffness, whereas cells adhered on Group IV SiNWs had the lowest stiffness (Fig. 3). While hMSCs grown on SiNWs groups with different stiffness, hMSCs received stress and stimulations and subsequently adapted to remodel and re-assemble their cytoskeleton, cell morphology, and gene expression^31–33^. After this adapting process, the stiffness of such differentiated cells is also affected by substrate stiffness^11–13,20,22,23,31–33^. The above results indicate that SiNWs with controllable spring constants are capable of regulating the osteogenicity and adipogenicity of hMSCs *in vitro*.

**Figure 4:**
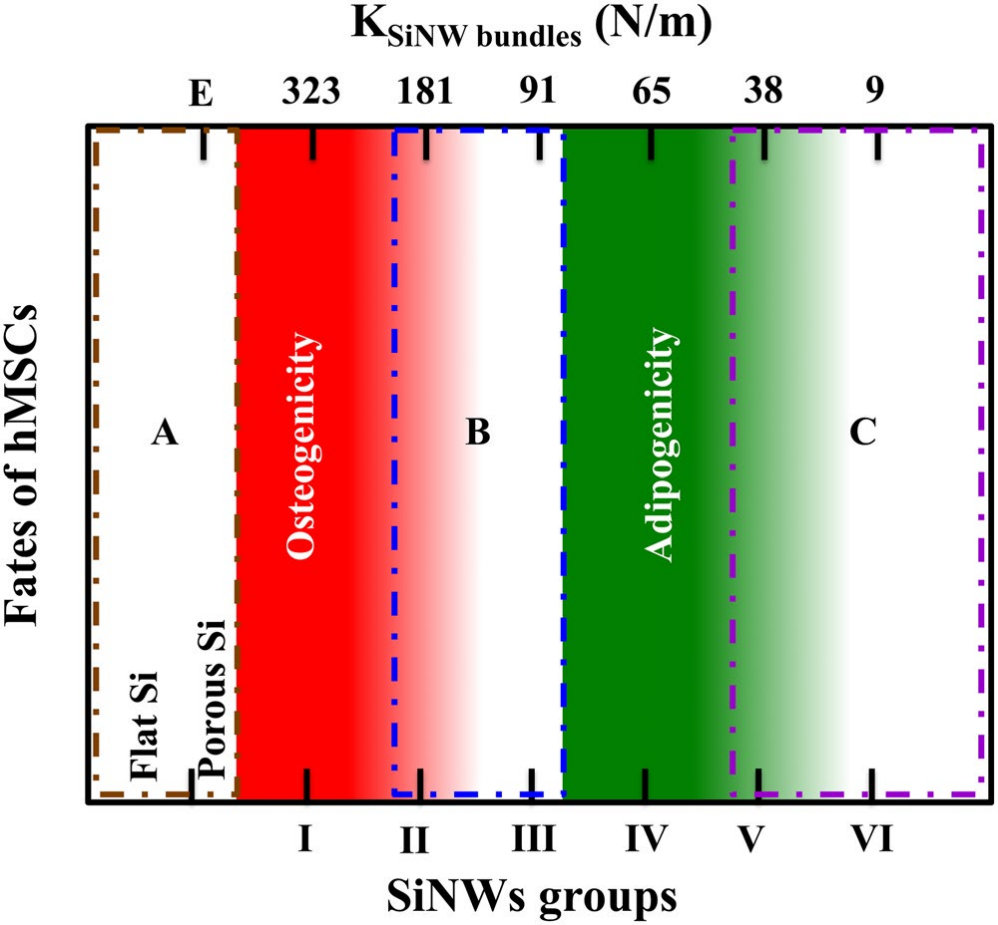
Fate-regulation map of hMSCs stimulated by the SiNWs with different dimensions and spring constants.

A fate-regulation map of the hMSCs is shown in Fig. 4. The Group I SiNWs (length = 9 μm; spring constant of SiNW bundles = 323 N/m) induced osteogenicity in the hMSCs, whereas the Group IV SiNWs (length = 26 μm; spring constant of SiNW bundles = 65 N/m) favored adipogenicity. The regions labeled A, B, and C in Fig. 4 indicate other possible fate regulations. According to the SiNW fabrication process from flat Si substrate, we divided this map into five regions. Group I and IV SiNWs represent osteogenicity and adipogenicity region, respectively. Region A means test samples did not perform EMD process (0 min, present as clean flat Si surface) or perform EMD process at very short period of time (<3 min, present as porous Si surface). In Region A, the mechanical property of the porous Si surface is similar to that of flat surfaces. However, unlike polymer based culture matrices, the porous and flat Si have very limited variations in their Young’s modulus^11,12^. This culture matrix in Region A has very low degree of freedom in substrate elasticity and may provide limited contributions to hMSCs differentiation. The spring constant of Group I SiNWs was nearly five times higher than that of Group IV SiNWs, and these SiNWs produced opposite differentiation results. Region B is the transition area between Group I and IV SiNWs. Region C is beyond Group V SiNWs. Unlike Group I and IV SiNWs, rest of the groups did not regulate hMSCs into obvious osteogenicity or adipogenicity. However, there might be some other possible differentiations of hMSCs in Region B and C. Like Group II and Group VI SiNWs, their spring constants were three times higher and seven times lower than that of the Group IV SiNWs, respectively, and might be potential candidates for directed other differentiations of hMSCs.

In summary, we successfully manipulated the spring constants of SiNWs, and the resulting SiNW matrices strongly supported hMSC adhesion, growth, and differentiation. The high-spring-constant SiNWs (Group I) induced osteogenicity in the hMSCs, whereas the low-spring-constant SiNWs (Group IV) favored adipogenicity. The gene expressions of osteogenic and adipogenic markers and cell stiffnesses were consistent with our hypothesis. This *in vitro* approach for the control of stem cell differentiation by SiNW matrices guarantees the consistent regulation of cell fate.

## Methods

### SiNW fabrication

A (100)-orientated Si single-crystal wafer was sliced into 1 × 1 cm^2^ pieces for SiNW fabrication, morphological observation, and hMSC cellular tests. Electroless metal deposition (EMD)^23,27^ was used to produce vertically aligned SiNW matrices. First, the Si chip was immersed in concentrated silver nitrate (AgNO_3_) solution for 10 min at 50 °C ± 1 °C to pre-deposit silver nanoparticles (AgNPs) as oxidizing agents onto the polished Si surface. Subsequently, we transferred the chips with AgNPs into the EMD electrolyte solution, which was composed of AgNO_3_/hydrofluoric acid (HF)/H_2_O. The EMD process was carried out at 50 °C ± 1 °C for different time periods to obtain uniform SiNW arrays: 5 min (Group I), 10 min (Group II), 15 min (Group III), 20 min (Group IV), 30 min (Group V), and 60 min (Group VI). Table S1, tabulated in the Supplementary information, lists the conditions for SiNW fabrication.

During the etching reaction, the pre-deposited AgNPs first oxidized the contact surface, leaving numerous tiny SiOx areas. These oxides were immediately etched away from the Si substrate by F^−^, forming many nano-pits on the surface. Much of the Ag^+^ from AgNO_3_ in the electrolyte was reduced into AgNPs on the Si surface; thus, the Si surface was continually oxidized, and HF continuously etched away the oxides. In this way, vertically aligned SiNWs were uniformly formed on the (100) Si single-crystal substrates. In addition, the huge amount of the concentrated AgNPs gradually transformed into Ag dendrites, which covered the entire sample. After the desired EMD reaction time, the samples were removed from the electrolyte solution, dipped in distilled water to stop the etching reaction, and transferred into HNO_3_ solution to dissolve the Ag dendrites.

These SiNWs samples were gently washed twice with distilled water to remove any residual EMD electrolyte and then dried on a hotplate at 120 °C for 10 min. Prior to cell culturing, the SiNW samples and flat Si chips were sterilized in a steam autoclave at 121 °C for 15 min and washed twice with PBS for 10 min.

### Evaluation of SiNW dimensions

The lengths and diameters of the SiNWs in all six groups were determined by SEM (JSM-6309, JEOL, Japan). The measurements were made for 300 individual SiNWs (n = 300), and their averages and standard deviations were reported. Based on the measured lengths and diameters, we used Hook’s law (Equation S1) and beam theory (Equation S2) to calculate the theoretical spring constants of the SiNWs (Equation 1):

S1 $$F=-\,kx,$$

and

S2 $$E=\frac{4{l}^{3}k}{w{t}^{3}},$$

where F is the applied force, K is the spring constant, x is the displacement, E is the Young’s modulus of flat (100) Si, l is the length of the object, w is the width of the object, t is the thickness of the object, and E is the Young’s modulus of (100) Si ( = 170 GPa), which is independent of dimensional factors. Beam theory is usually employed to calculate the spring constant of a cantilever; thus, l, w, and t are regarded as the dimensional factors of the cantilever. The samples in this study were cylindrical nanowires; thus, we replaced w and t with diameter D. Thus, to evaluate the theoretical spring constants of the SiNWs in this study, we transformed Equation S2 into Equation 1.

### SiNW characteristics

#### Determination of SiNW physical properties by *in situ* TEM picoindentation

We measured the actual SiNW spring constants by *in situ* TEM picoindentation using three pieces of equipment: a dual-beam focused ion beam (FIB) microscope (Nova-200 DBFIB NanoLab, FEI, US), a high-resolution TEM instrument (JEM-2010, JEOL, JAPAN), and a TEM picoindenter (PI-95 TEM Picoindenter, HYSITRON, US). Briefly, FIB was used to fabricate SiNW testing chips with dimensions of 10–30 μm (length) × 10–15 μm (width) × 1.5–2 μm (thickness). These chips were glued tightly on TEM Cu grids using an *in situ* Omniprobe for *in situ* TEM picoindentation.

Picoindentation was conducted using a flat-end cylindrical punch (contact area = 5 μm^2^) in front of top surface of the prepared SiNW sample fixed on the TEM holder. The indentation process was observed at an acceleration voltage of 200 KV. Indentation was performed to a depth of 400 nm, and the load, time, and displacement were recorded.

### Total RNA isolation, reverse transcription, and quantitative real-time polymerase chain reaction (RT-PCR)

A total of 1 × 10^5^ hMSCs were seeded onto SiNWs in 24-well plates in MesenPRO RS medium (GIBCO®, Invitrogen, Grand Island, NY, USA) incubated for 24 h. The cells were then transferred to adipogenic induction medium for 72 h. Total RNA was extracted using TRIzol reagent (Invitrogen). The messenger RNA in 2 μg total RNA was reverse transcribed to complementary DNA using MMLV High-Performance Reverse Transcriptase (EPICENTRE® Biotechnologies) followed by PCR amplification.

The gene expressions of hMSCs on the various SiNWs were detected by quantitative real-time polymerase chain reaction (qRT-PCR) using a StepOne PlusTM Real-time PCR System (Applied Biosystems). Intron spanning primers specific for each gene were designed using the Universal ProbeLibrary Assay Design Center and detected using the corresponding probes from the Universal ProbeLibrary (Roche Applied Sciences, Mannheum, Germany). The following primer and probe sequences were applied for qPCR: peroxisome proliferator-activated receptors gamma (*PPARγ*): forward primer 5′-GACAGGAAAGACAACAGACAAATC-3′, reverse primer 5′-GGGGTGATGTGTTTGAACTTG-3′, probe number 7; fatty acid binding protein 4 (*FABP4*): *RUNX-2* forward primer 5′-CTACCACCCCGCTGTCTTC-3′, reverse primer 5′-CAGAGGTGGCAGTGTCATCA-3′, probe number 29 and *COL1α1*: forward primer 5′ATGTTCAGCTTTGTGGACCTC3′, reverse primer 5′CTGTACGCAGGTGATTGGTG3′, probe number 15. The average threshold cycle (Ct) for each gene was normalized to that of GAPDH. The details of semi-qRT-PCR analysis are given in Table S2 in the Supplementary information.

### Elasticity measurements of hMSCs adhered on SiNWs

#### Immuno-staining of living hMSCs on SiNWs

For fixed cells, cells were fixated, dehydrated, and coated with Au–Pd for adherence to SiNWs. Cell morphology was fixed, and the cells could be observed by optical microscopy (OM) because of the strong reflection of the Au–Pd coatings. Consequently, we could determine the locations of the fixed hMSCs by OM-equipped AFM.

For living cells adhered to SiNWs in a liquid environment, the SiNW matrix is a promising anti-reflecting surface that absorbs more than 90% of light, and the liquid environment also inhibits observation. Thus, OM-equipped AFM is not suitable for determining the locations of living cells. Thus, we used an immune-staining approach to locate the living cells. The living cells and their locations were confirmed by staining with calcein acetoxymethyl ester (Calcein AM, Sigma-Aldrich, St. Louis, MO, USA), which appears green under fluorescence microscope. The adherent cells were stained with 2 μM Calcein AM for 30 min before measurement.

#### Elasticity measurements of fixed and living hMSCs on SiNWs

The elasticity of fixed hMSCs adhered on SiNWs was determined by AFM (Dimension Icon®, Bruker, USA) in contact mode in air phase with an SNL-10 tip (silicon tip on a nitride lever coated with 45-nm Ti–Au on the back side). The spring constant of the cantilever was 0.35 Nm^−1^, and its frequency was 50–80 kHz. Before indentation, polydimethylsiloxane was employed as a standard substrate to calibrate subsequent measurements. For fixed cells on SiNWs (dead cells), the SNL-10 tip was applied directly without sterilization. Randomly, 30–40 cells were indented in the central region to obtain their force curves for all SiNW groups.

In contrast, the elasticities of living cells adhered on SiNWs were measured using a Bioscope Catalyst instrument (Bioscope Catalyst, Bruker, USA) in peak force quantitative nanomechanical mapping mode was Advanced TEC- SPM-Sensor with tip at the end of the cantilever (ATEC-FM-10, without coating). The spring constant of the cantilever was 2.8932 Nm^−1^, its frequency was 85 kHz, its tip radius was 10 ± 2 nm, and its half angle was 12°. Prior to the indentation experiments, the AFM probes were cleaned and sterilized in ethanol to remove contaminants on the probe surface. The relationships between displacement and indentation of the cantilever in contact with the cells were obtained in force curves. In this study, the contact point was defined as the point at which the slope of the force curve was approximately zero. To determine the elasticity of living cells, 20 cells were measured for each SiNW group. The Young’s moduli were calculated using Sneddon fits^34^.

### Statistical analysis

One-way analysis of variance with Tukey’s post hoc tests was conducted using IBM SPSS 12.0 software. Differences considered to be statistically significant at P < 0.05 and <0.01 are demoted as b and c, respectively (b: P < 0.05; c: P < 0.01).

## Electronic supplementary material

Supplementary information
