# Supplementary material for: SiNWs Biophysically Regulate the Fates of Human Mesenchymal Stem Cells
Source: Sci Rep. 2018 Aug 27;8:12913. doi: 10.1038/s41598-018-30854-3 (PMC6110734; doi:10.1038/s41598-018-30854-3)
Supplement: Supplementary file 1 — Supplementary information [file 41598_2018_30854_MOESM1_ESM.docx]

SiNWs Biophysically Regulate the Fates of Human Mesenchymal Stem Cells

Hsin-I Lin^a,+^, Shu-Wen Kuo^b,+^, Ta-Jen Yen^a,d,e,*^, Oscar K. Lee^b,c,f,g,*^

^a^Department of Materials Science and Engineering, National Tsing Hua University, Hsinchu, Taiwan

^b^Department of Medical Research, Taipei Veterans General Hospital, Taipei, Taiwan

^c^Stem Cell Research Center, National Yang Ming University, Taipei, Taiwan

^d^Frontier Research Center on Fundamental and Applied Sciences of Matters, National Tsing Hua University, Hsinchu, Taiwan

^e^High Entropy Materials Center, National Tsing Hua University, Hsinchu, Taiwan

^f^Department of Orthopaedics and Traumatology, Taipei Veterans General Hospital, Taipei, Taiwan

^g^Taipei City Hospital, Taipei, Taiwan

Hsin-I Lin and Shu-Wen Kuo contributed equally in this work

^+^Co-first author

*Co-corresponding author

Professor Ta-Jen Yen

Department of Materials Science and Engineering,

National Tsing Hua University,

101 Sec. 2, Kuang-Fu Road,

Hsinchu 30013

Taiwan ROC

E-mail: tjyen@mx.nthu.edu.tw **Supplementary information**

1. **Methods**

| Table S1 Conditions of SiNW fabrication | | | | | | | |
| --- | --- | --- | --- | --- | --- | --- | --- |
| Group | | I | II | III | IV | V | VI |
| Pre-deposited AgNPs | AgNO_3_ concentration (M) | 0.065 | | | | | |
|  | Temperature (°C) | 50±1 | | | | | |
|  | Period (min) | 10 | | | | | |
| EMD reaction | Electrolyte | 0.03 M AgNO_3_+4.6 M HF | | | | | |
|  | Temperature (°C) | 50±1 | | | | | |
|  | Period (min) | 5 | 10 | 15 | 20 | 30 | 60 |

| Table S2 Primer sequences and probes from the Universal ProbeLibrary used in qRT-PCR analysis | | |
| --- | --- | --- |
| Gene name | Oligonucleotide sequence | Probe # |
| *RUNX-2* | 5′-CTACCACCCCGCTGTCTTC-3′  5′-CAGAGGTGGCAGTGTCATCA -3′ | 29 |
| *COL1α1* * | 5′-ATGTTCAGCTTTGTGGACCTC3′  5′-CTGTACGCAGGTGATTGGTG-3′ | 15 |
| *PPARγ* | 5′-GACAGGAAAGACAACAGACAAATC-3′  5′-GGGGTGATGTGTTTGAACTTG-3′ | 7 |
| *FABP4* | 5′-CCACCATAAAGAGAAAACGAGAG-3′  5′GTGGAAGTGACGCCTTTCAT3′ | 31 |
| * *COL1α1*: collagen type 1 alpha 1 | | |
